# Potential pitfalls of using a correction to normative values for the assessment of acid–base compensation during early ascent to high altitude

**DOI:** 10.1113/EP093321

**Published:** 2025-11-05

**Authors:** Nicole V. Bushfield, Trevor A. Day

**Affiliations:** ^1^ Center for Chronic Disease Prevention and Management, Faculty of Medicine University of British Columbia Kelowna British Columbia Canada; ^2^ Department of Biology, Faculty of Science and Technology Mount Royal University Calgary Alberta Canada; ^3^ Department of Physiology and Pharmacology, Cumming School of Medicine University of Calgary Calgary Alberta Canada

## AUTHOR CONTRIBUTIONS

Both authors wrote, reviewed, edited and approved the final manuscript and agree to be accountable for all aspects of the work in ensuring that questions related to the accuracy or integrity of any part of the work are appropriately investigated and resolved. All persons designated as authors qualify for authorship, and all those who qualify for authorship are listed.

## CONFLICT OF INTEREST

None declared.

## 1 INTRODUCTION

We read with interest the article by Skalla and colleagues recently published in *Experimental Physiology* (Skalla et al., 2026). The addition of data highlighting the early time course of respiratory and renal responses with ascent to a moderately high altitude contributes to our understanding of the early phases of integrated responses to acute exposure and acclimatisation in healthy humans with ascent.

Many organ systems respond to the acute hypoxic stimulus with high altitude ascent, and indeed, the response of one organ system (e.g., the hypoxic ventilatory response and resulting hypocapnia and respiratory alkalosis) is the stimulus for another (e.g., renal compensation via bicarbonate diuresis). Assessing the early time course of exposure to high altitude is of interest to applied human physiologists and clinicians alike, due to the dynamic nature of integrated acute responses and acclimatisation, and potential implications for acute mountain sickness.

Skalla et al. (2026) measured arterial blood gases and acid–base variables before and 24 and 44 h after arrival at 3100 m. We noted that despite an incremental reduction in [HCO_3_^−^]_a_, pH remained alkalaemic, even after 44 h at 3100 m. We were particularly pleased to see our ‘renal reactivity’ metric applied to early ascent, where each individual's renal response (Δ[HCO_3_^−^]_a_) was indexed against the renal stimulus, taken as Δ$P_{\mathrm{aC}O_{2}}$, following ascent (Zouboules et al., 2018).

We noted that Skalla et al. (2026) also assessed early blood acid–base acclimatisation using altitude‐corrected extracellular fluid base excess (ecf‐BE), mathematically equivalent to titratable hydrogen ion difference (THID), a correction initially introduced and intended for application in acclimatised and chronically dwelling high‐altitude residents (e.g., Zubieta‐Calleja et al., 2011).

Base excess (BE) is calculated via the Van Slyke equation as per Siggard‐Anderson (1977):


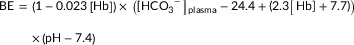

where [Hb] is [haemoglobin].

Titratable hydrogen ion difference (THID) as advanced in Zubieta‐Calleja et al. (2011):

$$\begin{array}{ccc} \mathrm{THID} & = & \left(1-\left[\mathrm{Hb}\right]/43\right)\times ([\mathrm{actual}\;\mathrm{HC}{O_{3}}^{-}]-[\mathrm{altitude}\mathrm{HC}{O_{3}}^{-}])\\ & & +\;\beta B\times \left(\mathrm{pH}-7.4\right) \end{array}$$

where [Hb] is [haemoglobin], [altitude HCO_3_^−^] = 24.32 – (1.8 × (altitude in km)), and βB represents the non‐bicarbonate buffer capacity of whole blood, calculated as 2.3[Hb] + 7.7.

With ascent to high altitude and renal compensation, the Van Slyke equation results in a negative BE (or, in other words, a base deficit), congruent with reduced arterial [bicarbonate], as expected. However, the THID equation takes into account the altitude, normalising BE toward assumed baseline values.

We have two main critiques regarding the use of this derivation of BE with early ascent, namely: (a) THID was not developed to assess early‐acclimatisation responses, but rather developed as a steady‐state tool to assess acid–base status with sustained high‐altitude residence, after which time acid–base homeostasis has stabilized, and (b) the use of assumed normative values in these correction factors is potentially misleading, particularly when researchers have each participant's actual measured values available.

## 2 THE DEVELOPMENT AND USE OF THID

THID (ecf‐BE) is a steady‐state normalisation technique originally intended to assess acid–base status in acclimatised high‐altitude residents. It was introduced to prevent erroneous diagnosis of acid–base disturbances in high altitude residents whose ventilatory and renal responses or compensation established a new steady‐state setpoint, in other words, a stable homeostatic target for $P_{\mathrm{aC}O_{2}}$, [HCO_3_^−^]_a_ and pH_a_ under chronic exposure and residence in the context of hypobaric hypoxia (Zubieta‐Calleja et al., 2011).

Accordingly, the original Van Slyke equation was modified so that individuals could be assessed at this new altitude‐acclimatised setpoint, allowing clinicians to distinguish a newly‐imposed (i.e., clinical) acid–base disturbance from the resident's ‘normal’ altitude‐compensated baseline (Zubieta‐Calleja et al., 2011). Thus, THID was developed in high altitude resident populations (e.g., indigenous highlanders in Bolivia) and assumes population‐level reference values for $P_{\mathrm{aC}O_{2}}$, [HCO_3_^−^]_a_ and pH_a_ consistent with full (or near‐full) renal compensation at that particular altitude (Zubieta‐Calleja et al., 2011).

## 3 ASSUMPTION OF FULL RENAL COMPENSATION

This specific clinical application does not translate to lowlanders during the first 24–44 h of ascent, a non‐steady‐state period, during which lowlanders typically remain alkalaemic for days (e.g., Zouboules et al., 2018), if not indefinitely. Applying correction factors to visitors can (a) normalise away the very perturbation we seek to quantify with acute ascent, namely, acute respiratory alkalosis with early but incomplete renal compensation, and (b) invert or obscure the direction and magnitude of these responses. In this non‐steady‐state time domain, marked by substantial within‐ and between‐individual variability (e.g., Zouboules et al., 2018), using a correction anchored to assumed ‘textbook’ values is unnecessary when each participant's $P_{\mathrm{aC}O_{2}}$, [HCO_3_^−^]_a_ and pH_a_ are measured directly, and it risks distorting the interpretation of inter‐individual variability in response magnitudes.

When describing physiological responses in this early time domain of high‐altitude ascent, the critical information is the within‐individual trajectory (Δ$P_{\mathrm{aC}O_{2}}$, Δ[HCO_3_^−^]_a_ and ΔpH_a_), as Skalla et al. (2026) also address. THID/ecf‐BE was designed for the clinical problem of misclassification of acid–base disorders in high‐altitude residents who are persistently alkalaemic, despite reduced bicarbonate (with associated negative BE values), not for quantifying acute physiology in high‐altitude sojourners, and accordingly, in our view, should not be used to infer rapid renal compensation over 24–44 h, as in Skalla et al. (2026).

## 4 UTILISING NORMATIVE POPULATION‐LEVEL VALUES

THID/ecf‐BE and related corrections, like the original BE calculation using the Van Slyke equation, rely on population‐level reference values for $P_{\mathrm{aC}O_{2}}$, [HCO_3_^−^]_a_ and pH_a_. In the first 24–44 h after ascent to high altitude, where both baseline (pre‐ascent) and post‐ascent response values are heterogeneous, applying a single assumed reference value can distort the signal when each participant's $P_{\mathrm{aC}O_{2}}$, [HCO_3_^−^]_a_ and pH_a_ have been measured directly. In this early time domain, the variables of interest are the within‐individual change from baseline (Δ$P_{\mathrm{aC}O_{2}}$, Δ[HCO_3_^−^]_a_ and ΔpH_a_), not proximity to any external average population reference set.

Reference value choice also introduces biological bias. For example, baseline $P_{\mathrm{aC}O_{2}}$ and [HCO_3_^−^]_a_ differ by sex and menopausal status, as premenopausal females typically exhibit reduced $P_{\mathrm{aC}O_{2}}$ and [HCO_3_^−^]_a_ compared with postmenopausal females and males (e.g., Loeppky et al., 2001), so using a single assumed reference value to calculate a correction index will systematically bias the reporting of female responses, and can inflate apparent ‘compensation’.

Population heterogeneity further limits the utility of generalised correction factors. Indigenous highlanders are not interchangeable; Andean and Sherpa groups demonstrate distinct acid–base profiles across comparable altitudes (e.g., Tymko et al., 2022), and recent data demonstrate lowlanders differ from Tibetan highlanders with ascent and acclimatisation (e.g., Johnson et al., 2025). Consequently, applying a population‐specific, Bolivian‐derived correction factor to non‐Bolivian highland populations or lowlanders with early ascent assumes a uniformity that does not exist. In addition, we recently showed that Tibetan highlanders have differential $P_{CO_{2}}$ and [HCO_3_^−^] compared to lowlanders even at 1400 m (Bushfield et al., 2025), so there is appreciable variability within and between groups, even prior to ascent.

## 5 CONCLUSIONS

We appreciate Skalla and colleagues’ careful staging and early time course measurements with ascent to ∼3100 m. The dataset is valuable for adding to our emerging understanding of integrated respiratory–renal responses and elucidating interactions between organ systems over the first 2 days of acclimatisation to moderately high altitude. We suggest centring the presentation of data on participant‐level trajectories of the biochemical variables of interest, namely, $P_{\mathrm{aC}O_{2}}$, [HCO_3_^−^]_a_ and pH_a_, where the conclusions do not depend on a correction to a single normative reference set. We offer these suggestions on the potential pitfalls of correction factors and hope that this discussion sharpens physiological inferences drawn for this and other contributions to the literature.

## References

[eph70082-bib-0001] Bushfield, N. V. , Johnson, N. A. , Dickenson, J. A. , Mackenzie, B. W. , Isakovich, R. , Kalker, A. , Bouten, J. , Strzalkowski, N. D. J. , Harman, T. S. , Holmström, P. , Kunwar, A. J. , Thakur, N. , Dhungel, S. , Sherpa, N. , Bigham, A. , Brutsaert, T. D. , & Day, T. A (2025). No altitude required: Differential ventilatory and renal acid‐base homeostasis between unacclimatized lowlanders and Tibetan highlanders at 1,400 m. Journal of Applied Physiology, 139(4), 1064–1072. 40965521 10.1152/japplphysiol.00619.2025

[eph70082-bib-0002] Johnson, N. A. , Dickenson, J. A. , MacKenzie, B. W. L. , Isakovich, R. , Kalker, A. , Bouten, J. , Strzalkowski, N. D. J. , Harman, T. S. , Holmström, P. , Kunwar, A. J. , Thakur, N. , Dhungel, S. , Sherpa, N. , Bigham, A. W. , Brutsaert, T. D. , & Day, T. A (2025). Comparing integrative ventilatory and renal acid‐base acclimatization in lowlanders and Tibetan highlanders during ascent to 4,300 m. Proceedings of the National Academy of Sciences of the United States of America, 122(1), e2412561121. 39793031 10.1073/pnas.2412561121 PMC11725942

[eph70082-bib-0003] Loeppky, J. A. , Scotto, P. , Charlton, G. C. , Gates, L. , Icenogle, M. , & Roach, R. C (2001). Ventilation is greater in women than men, but the increase during acute altitude hypoxia is the same. Respiration Physiology, 125(3), 225–237. 11282389 10.1016/s0034-5687(00)00221-8

[eph70082-bib-0004] Siggaard‐Andersen, O. (1977). The van Slyke equation. Scandinavian journal of clinical and laboratory investigation. Supplementum, 146, 15–20. 13478 10.3109/00365517709098927

[eph70082-bib-0005] Skalla, E. , Treml, B. , Rajsic, S. , Schreinlechner, M. , Bukumirić, Z. , Berek, K. , Egger, A. , Knotzer, J. , Burtscher, M. , & Kleinsasser, A. (2026). Early acclimatization to high altitude: Acid–base and fluid balance dynamics during the first 2 days at 3100 m. Experimental Physiology, 111(3), 1387–1396. 10.1113/EP093029 PMC12949202 40853906

[eph70082-bib-0006] Tymko, M. M. , Willie, C. K. , Howe, C. A. , Hoiland, R. L. , Stone, R. M. , Tymko, K. , Tymko, C. , MacLeod, D. , Anholm, J. D. , Gasho, C. , Villafuerte, F. , Vizcardo‐Galindo, G. , Figueroa‐Mujica, R. , Day, T. A. , Bird, J. D. , Foster, G. E. , Steinback, C. D. , Brugniaux, J. V. , Champigneulle, B. , … Ainslie, P. N. (2022). Acid‐base balance at high altitude in lowlanders and indigenous highlanders. Journal of Applied Physiology, 132(2), 575–580. 35023761 10.1152/japplphysiol.00757.2021

[eph70082-bib-0007] Zouboules, S. M. , Lafave, H. C. , O'Halloran, K. D. , Brutsaert, T. D. , Nysten, H. E. , Nysten, C. E. , Steinback, C. D. , Sherpa, M. T. , & Day, T. A (2018). Renal reactivity: Acid‐base compensation during incremental ascent to high altitude. The Journal of Physiology, 596(24), 6191–6203. 30267579 10.1113/JP276973 PMC6292812

[eph70082-bib-0008] Zubieta‐Calleja, G. , Zubieta‐Castillo, G. , Zubieta‐Calleja, L. , Ardaya‐Zubieta, G. , & Paulev, P. E (2011). Do over 200 million healthy altitude residents really suffer from chronic acid‐base disorders?. Indian Journal of Clinical Biochemistry, 26(1), 62–65. 22211016 10.1007/s12291-010-0088-9 PMC3068777

